# Host Phylogeny Determines Viral Persistence and Replication in Novel Hosts

**DOI:** 10.1371/journal.ppat.1002260

**Published:** 2011-09-22

**Authors:** Ben Longdon, Jarrod D. Hadfield, Claire L. Webster, Darren J. Obbard, Francis M. Jiggins

**Affiliations:** 1 Institute of Evolutionary Biology, University of Edinburgh, Ashworth Labs, Edinburgh, United Kingdom; 2 Centre for Immunity, Infection and Evolution, University of Edinburgh, Ashworth Labs, Edinburgh, United Kingdom; 3 Department of Genetics, University of Cambridge, Cambridge, United Kingdom; Stanford University, United States of America

## Abstract

Pathogens switching to new hosts can result in the emergence of new infectious diseases, and determining which species are likely to be sources of such host shifts is essential to understanding disease threats to both humans and wildlife. However, the factors that determine whether a pathogen can infect a novel host are poorly understood. We have examined the ability of three host-specific RNA-viruses (*Drosophila* sigma viruses from the family *Rhabdoviridae*) to persist and replicate in 51 different species of Drosophilidae. Using a novel analytical approach we found that the host phylogeny could explain most of the variation in viral replication and persistence between different host species. This effect is partly driven by viruses reaching a higher titre in those novel hosts most closely related to the original host. However, there is also a strong effect of host phylogeny that is independent of the distance from the original host, with viral titres being similar in groups of related hosts. Most of this effect could be explained by variation in general susceptibility to all three sigma viruses, as there is a strong phylogenetic correlation in the titres of the three viruses. These results suggest that the source of new emerging diseases may often be predictable from the host phylogeny, but that the effect may be more complex than simply causing most host shifts to occur between closely related hosts.

## Author Summary

Emerging infectious diseases such as SARS, HIV and swine-origin influenza have all been recently acquired by humans from other species. Understanding the reasons why parasites jump between different host species is essential to allow us to predict future threats and understand the causes of disease emergence. Here we ask how host-relatedness might determine when host-shifts can occur in the most important group of emerging diseases—RNA viruses. We show that the relationship between host species is the primary factor in determining a virus's ability to persist and replicate in a novel host following exposure. This can be broken down into two components. Firstly, species closely related to the virus's natural host are more susceptible than distantly related species. Secondly, independent of the distance effect, groups of closely related host species have similar levels of susceptibility. This has important implications for our understanding of disease-emergence, and until now the only large-scale studies of viruses have been correlative rather than experimental. We also found groups of related species that are susceptible to these viruses but are distantly related to the natural hosts, which may explain why viruses sometimes jump between distantly related species.

## Introduction

A major source of emerging infectious diseases are host shifts, where the parasite originates from a different host species. In humans, HIV [1], influenza [2] and *Plasmodium* [3] have all been recently acquired from other species. Host shifts can also have devastating effects on wildlife; for example Ebola epidemics have resulted in marked declines in some primate populations [4] and canine distemper virus has jumped from dogs into Serengeti lions and caused considerable mortality [5]. As we have come to realise that the sources of human, domestic animal or crop pathogens are likely to be from wild species [6], [7], understanding what causes these parasite host shifts to occur has become increasingly important.

For a host shift to occur, the new host must first be exposed to the parasite, the parasite must then be able to replicate in the new host, and finally there must be sufficient onward transmission in the new host for the infection to spread in the population [6]. Exposure is clearly important in determining whether a host shift occurs, and some cases of disease emergence have followed changes in the geographic range of species that have brought parasites in contact with new hosts [5], [8], [9], [10]. However, once exposure has occurred, the factors that determine whether the pathogen can replicate in a new host are poorly understood.

One factor that can potentially affect whether a parasite can replicate in a new host species is host relatedness — parasites may be more likely to replicate in species closely related to the original host [11], [12], because closely related hosts will tend to present a more similar environment to the parasite. Parasites must evade an elaborate array of host defences and rely on the host for their physiological needs, and this will result in specialised adaptations [13], [14]. These adaptations have in turn resulted in some extremely specialised parasites that are only able to survive in a narrow range of similar host species [15]. If this is the case, host shifts may occur most frequently between closely related species.

Here we use a new analytical approach to analyse host shifts, which allows us to separate two different ways in which the host phylogeny might affect the ability of a parasite to infect a new host species. The first of these, which we term the ‘distance effect’, reflects the fact that the chances of successful infection may be higher in species that are more closely related to the natural host. However, it is also likely that related species share similar levels of susceptibility independently of how related they are to the natural host, a process that we term the ‘phylogenetic effect’. These are statistically and biologically distinct phenomena. The distance effect will result in the expected susceptibility of new hosts declining as they become less related to the natural host. In contrast, the phylogenetic effect will have no effect on the expected susceptibility with distance from the natural host. However, it will result in distantly related species often having very different levels of susceptibility from the natural host, as it results in the variance in susceptibility increasing among more distantly related species.

The two effects may generate very different patterns of host switching. The distance effect would result in most host shifts infecting species closely related to the natural host. In contrast, the phylogenetic effect might mean that host clades distantly related to the natural host are susceptible to a parasite, and this could cause parasites to jump between distantly related species.

Previous research has examined the distance effect only. While there is evidence that parasites most often shift between related hosts from correlative studies of parasite-incidence in wild animals (e.g. [16]), experimental evidence has been surprisingly rare. Cross-infection experiments using plants and fungi [17], [18], *Drosophila* and nematode worms [19], and beetles and *Spiroplasma* bacteria [20] have all found that the ability of a parasite to establish an infection declines as a novel host's relatedness to the natural host declines.

The extent to which host relatedness influences host switching varies between different groups of parasites, and it has been suggested that RNA viruses may be particularly prone to jump between distantly related hosts [21]. Reviewing emerging viral diseases in vertebrates, Parrish *et al* [21] observed that “Spillover or epidemic infections have occurred between hosts that are closely or distantly related, and no rule appears to predict the susceptibility of a new host.” Viruses are more likely than other groups of parasites to be shared between distantly related primates [16], and many human diseases that have been recently acquired from other species are RNA viruses [22]. The ability of certain viruses to infect distantly related hosts may result from the use of conserved host receptors to enter cells [23], [24], or the existence of hosts that do not posses broad resistance mechanisms to that type of parasite [25], [26]. However, some studies have found evidence for the importance of the host phylogeny; rabies virus strains have higher rates of cross species transmission between closely related host species in the wild [27] and primate lentivirus phylogenies show signs of preferential switching between closely related hosts [28].

To explore this question we have conducted a large cross-infection experiment in which three sigma viruses were injected into 51 different species of Drosophilidae. Sigma viruses are a clade of rhabdoviruses (RNA viruses with single-stranded negative-sense genomes), which infect various species of Diptera [29], [30]. They are normally vertically transmitted [31], [32], leading to extreme specialisation on just a single host species. However, the sigma virus of *Drosophila melanogaster* (DMelSV) will replicate in a range of different dipteran hosts [33], and differences between the host and virus phylogenies show that sigma viruses have switched between distantly related host lineages during their evolution [30]. Here we find that the host phylogeny explains most of the variation in the ability of sigma viruses to replicate in novel hosts, with both the distance and phylogenetic effects being large. These results not only allow us to explore the different ways in which the host phylogeny may affect host switching, but they are also, to our knowledge, the first study to experimentally test the effect of host genetic distance on infection success in RNA viruses — the most important source of emerging diseases.

## Materials and Methods

**Figure 1:**
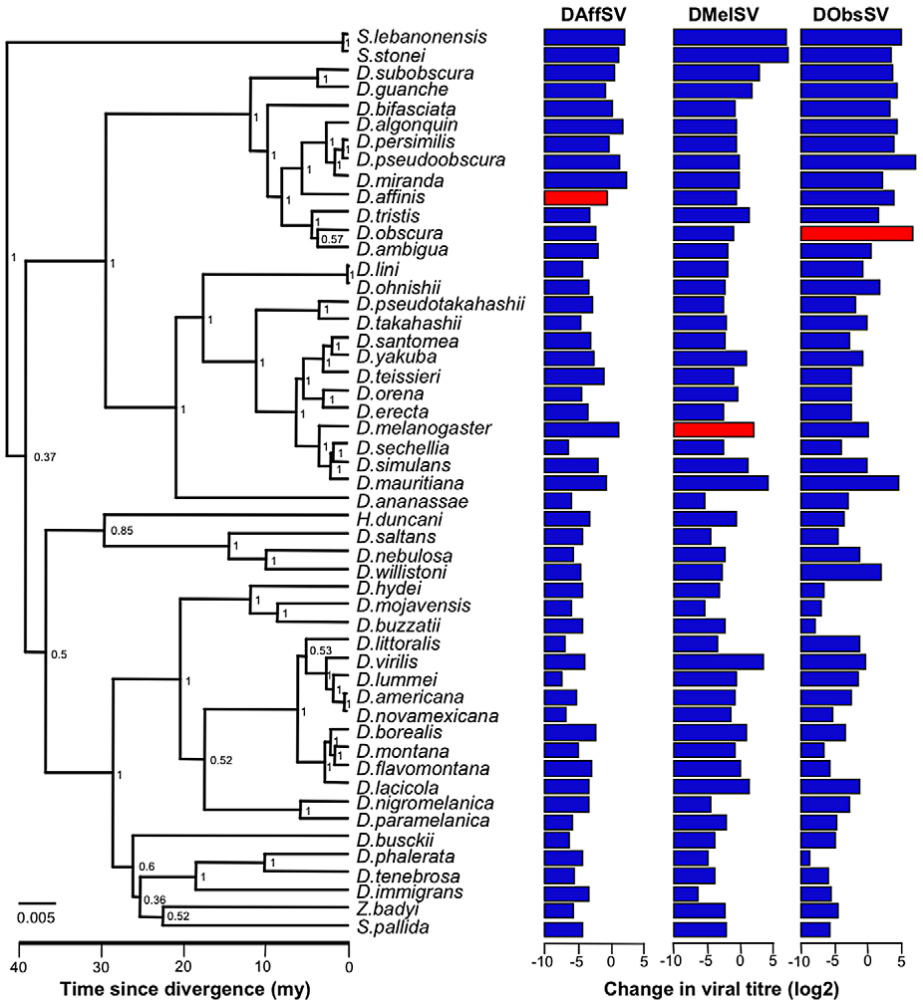
Phylogeny of host species and the respective mean change in viral titre (log2 scale) for each species-virus combination. Natural host-virus combinations are in red. The phylogeny was inferred under a relaxed molecular clock, node labels are posterior supports, the scale bar is number of substitutions per site and the scale axis represents the approximate age since divergence in millions of years (my) based on estimates from [60].

We measured the ability of three *Drosophila* sigma viruses to persist and replicate following injection into 51 fly species sampled from across the phylogeny of the Drosophilidae (Figure 1). The three viruses were DAffSV, DMelSV and DObsSV, which naturally occur in *D. affinis, D. melanogaster* and *D. obscura* respectively [29].

### Virus isolates

We extracted DAffSV, DMelSV and DObsSV from infected stocks of *D. affinis, D. melanogaster* and *D. obscura.* To clear these stocks of any bacterial or other viral infections, they were aged for at least 20 days, before collecting embryos [32] and de-chorionating them in ∼2.5% w/v sodium hypochlorite solution for one minute [31]. The embryos were then rinsed in distilled water and placed onto clean food. To collect flies infected with a sigma virus, the adults were exposed to 100% CO_2_ at 12°C for 15 mins and the paralysed individuals were retained [31], [32], [34]. These were frozen at −80°C to rupture cells, homogenised in Ringer's solution [35] (2.5 µl/fly), and then briefly centrifuged twice, each time retaining the supernatant. This was passed through Millex PVDF 0.45 µM and 0.22 µM syringe filters (Millipore, Billerica, MA, USA) to remove any remaining host cells or bacteria, before being stored in aliquots at −80°C.

### Injections

Stocks of each fly species were kept in half pint bottles of staggered ages, and each day freshly eclosed flies were sexed, males were removed, and females were aged at 18°C for 3 days on agar medium (recipe in Text S1) before injection. At the same time we stored remaining flies in ethanol for wing size measurements. The food medium, rearing temperature and whether each species was composed of single or multiple lines can be found in Table S1.

Female flies were injected with 69 nl of the virus extract intra-abdominally using a Nanoject II micro-injector (Drummond scientific, Bromall, PA, USA). Half the flies were frozen immediately in liquid nitrogen as a reference sample to control for relative dose size, and the rest were kept on agar medium at 18°C for 15 days to allow the virus to replicate before being frozen in liquid nitrogen. The day 15 time-point was chosen based on pilot time-course data, and we note that the change in viral titre includes a decline in the virus following injection, followed by a growth/replication phase (Figure S1). Frozen flies were then homogenised in Trizol reagent (Invitrogen Corp, San Diego, CA, USA). Based on quantitative reverse-transcription PCR (qRT-PCR), the dose of the three viruses was similar (with a maximum of a 1.6x difference between viruses).

The injections were carried out over a period of 18 days, with the aim of completing 3 biological replicates for each virus per fly species (3 replicates each of the day 0 and day 15 treatments). The virus (DAffSV, DMelSV or DObsSV) was rotated on a daily basis, whilst treatment (frozen immediately or on day 15) and the injection order of fly species were randomised each day. On average we injected and quantified viral titre in a pool of 10 flies per replicate (range of across species means = 5–15). Out of the 153 fly-virus combinations, 126 had 3 biological replicates, 24 had 2 biological replicates and 3 had 1 biological replicate.

### Other factors

*Wolbachia* endosymbionts have recently been shown to provide resistance to a range of positive sense RNA viruses [36], [37], [38], [39]. Although it does not affect the replication of DMelSV (L. Wilfert and M. Magwire, unpublished data), we nonetheless tested each species for *Wolbachia* using PCR primers that amplify the *wsp* gene [40].

We also checked that the body size of the different species did not affect our results. To do this, we measured wing length, which is commonly used as a body size measure in *Drosophila* and strongly correlates with thorax length [41], [42]. Wings were removed from ethanol-stored flies, photographed under a dissecting microscope and the length of the IV longitudinal vein from the tip of the proximal segment to where the distal segment joins vein V [43] was measured (relative to a standard measurement) using ImageJ software (v1.43u) [44].

### Measuring change in viral titre

Viral titres were estimated using qRT-PCR. To ensure that we only amplified viral genomic RNA and not messenger RNA, the PCR primers were designed to amplify a region spanning two different genes. The copy-number of viral genomic RNA was expressed relative to the endogenous control housekeeping gene *RpL32* (*Rp49*). We designed different *RpL32* primers specific for each species. First, we sequenced the *RpL32* gene from all of the species (we were not able to amplify *RpL32* from *Drosophila busckii*, see Table S2). We then designed species-specific primers in two conserved regions (Table S3).

Total RNA was extracted from our samples using Trizol reagent, reverse-transcribed with Promega GoScript reverse transcriptase (Promega Corp, Madison, WI, USA) and random hexamer primers, and then diluted 1∶4 with DEPC treated water. The qRT-PCR was performed on an Applied Biosystems StepOnePlus system using a Power SYBR Green PCR Master-Mix (Applied Biosystems, CA, USA) and 40 PCR cycles (95°C for 15 sec followed by 60°C for 1 min). Two qRT-PCR reactions (technical replicates) were carried out per sample with both the viral and endogenous control primers. Each qRT-PCR plate contained a standard sample, and all experimental samples were split across plates in a randomised block design. A linear model was used to correct for the effect of plate. We repeated any samples where the two technical replicates had cycle threshold (*Ct*) values more than 1.5 cycles apart after the plate correction.

To estimate the change in viral titre, we first calculated Δ*Ct* as the difference between the cycle thresholds of the sigma virus qRT-PCR and the endogenous control. The viral titre of day 15 flies relative to day 0 flies was then calculated as 2^−ΔΔ*Ct*^ *,* where ΔΔ*Ct*  =  Δ*Ct_day0_* – Δ*Ct_day15_,* where Δ*Ct_day0_* and Δ*Ct_day15_* are a pair of Δ*Ct* values from a day 0 biological replicate and a day 15 biological replicate for a particular species-virus combination. We used a dilution series to calculate the PCR efficiency of the three sets of viral primers and thirteen of the *RpL32* primer combinations (covering 40 of the 51 *Drosophila* species). The efficiencies of the three virus primers were 95%, 97%, and 100%, (DAffSV, DMelSV and DObsSV) and the average efficiency of *RpL32* primers across species was 106%, with all being within a range of 98–112%.

### Host phylogeny

The host phylogeny was inferred using the *COI, COII, 28S rDNA, Adh, SOD, Amyrel* and *RpL32* genes. We downloaded all the available sequences from Genbank, and attempted to sequence *COI, COII, 28S rDNA, Adh* and *Amyrel* in those species from which they were missing (details in Table S4). This resulted in sequence for all species for *COI, COII* and *28S* and partial coverage for the other genes (50 out of 357 species-locus combinations were missing from the data matrix). The sequences of each gene were aligned using ClustalW (alignments and accession numbers are Datasets S1-S8 in supporting information). To reconstruct the phylogeny we used BEAST [45], as this allows construction of an ultrametric (time-based) tree using a relaxed molecular clock model. The genes were partitioned into 3 groups each with their own substitution and molecular clock models. The three partitions were: mitochondrial (*COI, COII*); ribosomal (*28S*); and nuclear (*Adh, SOD, Amyrel, RpL32*). Each of the partitions used a HKY substitution model (which allows transitions and transversions to occur at different rates) with a gamma distribution of rate variation with 4 categories and estimated base frequencies. Additionally the mitochondrial and nuclear data sets were partitioned into codon positions 1+2 and 3, with unlinked substitution rates and base frequencies across codon positions. Empirical studies suggest that HKY models with codon partitions are a good fit for most protein coding data sets [46]. A random starting tree was used, with a relaxed uncorrelated lognormal molecular clock and we used no external temporal information, so all dates are relative to the root age. The tree-shape prior was set to a speciation-extinction (birth-death) process. The BEAST analysis was run for 100 million MCMC generations sampled every 1000 steps (additionally a second run was carried out to ensure convergence). The MCMC process was examined using the program Tracer (v1.4) [47] to ensure convergence and adequate sampling. Trees were visualised using FigTree (v. 1.3) [48].

### Statistical analysis

We used a phylogenetic mixed model to examine the effects of host relatedness on viral persistence and replication in a new host [49], [50], [51]. This framework allows (random) phylogenetic effects to be included in the model, with the correlation in phylogenetic effects between two host species being inversely proportional to the time since those two host species shared a common ancestor (following a Brownian model of evolution). In general, conclusions drawn from phylogenetic comparative methods that include a species term in the model seem to be robust to alternative (non-Brownian) evolutionary models [52].

We fitted the model using a Bayesian approach in the R package MCMCglmm [53, R Foundation for Statistical Computing, Vienna, Austria] and REML in ASReml [54]. The two methods gave similar results so we only report the Bayesian analysis (Figure S2). The model had the form:
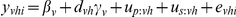
where *y_vhi_* is the viral titre of the *i^th^* biological replicate of host species *h* infected with virus *v*. 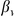 is the intercept term for virus *v*, and can be interpreted as the viral replication rate in the species at the root of the phylogeny. *d_vh_* is the phylogenetic (patristic) distance between the original host of virus *v* and species *h*, and the associated regression coefficient (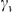) determines the degree to which viral replication rate of virus *v* changes as the phylogenetic distance increases. The random effect *u_p:vh_* is the deviation from the expected viral replication rate for virus *v* in host *h* due to historical processes (i.e. the host phylogeny). The species random effect *u_s:vh_* is the deviation from the expected viral replication rate of virus *v* in host *h* that is not accounted for by the host phylogeny. The residual is *e_vhi,_* which included within-species genetic effects, individual and micro-environment effects and measurement/experimental error. The random effects (including the residual) are assumed to come from multivariate normal distributions with zero mean vectors (because they are deviations) and structured covariance matrices. Denoting 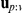
as a vector of phylogenetic effects across species for virus *v*, and **A** as a matrix with elements *a_jk_* representing the proportion of time that species *j* and *k* have had shared ancestry since the root of the phylogeny:
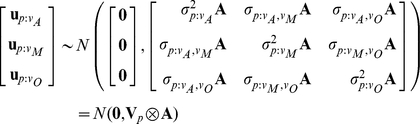
where 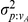 is the variance of phylogenetic effects for DAffSV, and 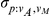 is the covariance between phylogenetic effects for DAffSV and DMelSV.

Similar distributions are assumed for species effects:
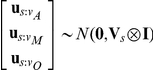
where **I** is an identity matrix indicating that species effects are independent of each other. The posterior modes for 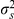
were close to zero for viruses DAffSV and DObsSV and these were omitted from the model (except for the calculation of σ^2^ *_p_/*(σ^2^ *_p_*+ σ^2^ *_s_*), see below).

The residuals are distributed as:
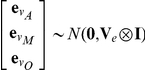
The off-diagonal elements of **V**_e_ (i.e. the covariances) were set to zero since viruses were not replicated within biological replicates. In a Bayesian analysis prior probability distributions have to be specified for the fixed effects and the covariance matrices. As described in detail in the supporting materials (Text S1) we used several different priors to check if the results are sensitive to the choice of prior. The results presented were obtained using parameter expanded priors for the **V**_p_ and **V**_s_ matrices [53]. The *P*-values reported (*P_MCMC_*) correspond to 2*p_min_*, where *p_min_* is the smaller of the two quantities a) the proportion of iterations in which the posterior distribution is positive or b) the proportion of iterations in which the posterior distribution is negative. The 95% credible intervals (CI) were taken to be the 95% highest posterior density intervals. Marginal means of the posterior distribution are used as summaries of central tendency. Significance of the fixed effects was inferred if the 95% CI of the posterior distribution did not cross zero, and the *P*-values were equal to or less than 0.05.

We also checked whether several additional factors affected viral replication by repeating the analysis with these factors included in the model as fixed effects. There was no significant effect of wing size (an average of 33 measured per species, *P_MCMC_* = 0.50), the presence of the bacterial endosymbiont *Wolbachia* (Table S4, *P_MCMC_*  = 0.51) or rearing temperature (*P_MCMC_*  = 0.55). We also repeated the analysis with three outliers removed, so that the distribution of the residuals was not significantly different from normal according to an Anderson-Darling test (*A* = 0.61, *P* = 0.11). The parameter estimates were very similar to those obtained when including all the taxa (as reported in the results).

## Results

We measured the change in viral titre over 15 days for three sigma viruses each injected into 51 species of *Drosophila*, including their natural hosts (see Figure 1). In total we injected and quantified viral titre in 887 biological replicates (a total of 8762 flies). To investigate how the host phylogeny affects the ability of the virus to persist and replicate in the different species, we reconstructed the phylogeny of all 51 species using the sequences of seven different genes. The resulting tree broadly corresponds to previous studies [55], [56], with the close phylogenetic relationships being generally well supported and more ancient nodes were less well supported (Figure 1).

There are two ways in which the host phylogeny could affect the ability of the three viruses to infect new host species. First, the chances of successful infection may be higher in species that are more closely related to the natural host (the ‘distance effect’). Second, related species may share similar levels of susceptibility independently of how related they are to the natural host — an effect that we refer to as the ‘phylogenetic effect’. To separate these two processes we fitted a phylogenetic mixed model to our data.

**Figure 2:**
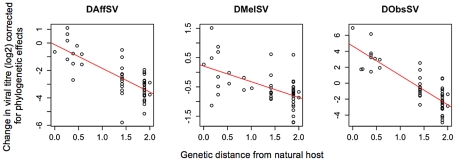
The effect of the genetic distance of a novel host from the natural host on the titre of three sigma viruses 15 days after injection. The estimates of viral titre have been corrected for phylogenetic effects and are plotted on a log2 scale. Genetic distance is relative to the distance from root to tip (root to tip equals 1). Trend line is for illustrative purposes.

All three viruses have greater viral titres in fly species that are more closely related to their natural host (Figure 2). If we assume that titres of all three viruses decline with genetic distance from their natural host at the same rate, then there is a significant negative relationship between titre and distance (slope: γ  =  −1.96; 95% CI =  −3.66, −0.43; *P_MCMC_*  = 0.022). If we instead allow the effect to differ between viruses, the negative effect of genetic distance from the natural host on replication is greatest for DObsSV (Figure 2; slope: γ*_O_*  =  −4.03; 95% CI  = −6.11, −0.94; *P_MCMC_*  = 0.005), is smaller and only marginally non-significant for DAffSV (Figure 2; slope: γ*_A_*  =  −1.82; 95% CI  = −3.99, 0.37; *P_MCMC_*  = 0.095), and not significant for DMelSV (Figure 2; slope: γ*_M_*  =  −0.47; 95% CI  = −3.06, 1.94; *P_MCMC_*  = 0.692). These effects were still present when the natural host species were removed from the analysis (data not shown). Therefore, the rate at which viral titres decline with genetic distance of the new host from the natural host differs between the individual viruses.

There is also a strong influence of host phylogeny on viral replication that could not be explained by the distance of the novel host from the original host. The between-species variance consists of two components; σ^2^ *_p_*, which is the variance that can be explained by the host phylogeny, and a species-specific component σ^2^ *_s_* which cannot be explained by a Brownian-motion model of evolution on the host phylogeny. These statistics do not include the effects of the distance from the natural host, as this was included as a fixed effect in the model [57]. To assess the importance of the host phylogeny, we calculated the proportion of the between-species variance that can be explained by the phylogeny (σ^2^ *_p_/*(σ^2^ *_p_*+ σ^2^ *_s_*), which is similar to Pagel's λ [58], [59] or phylogenetic heritability [50], [51]). The phylogeny explained almost all of the between-species variance in viral titre for DAffSV and DMelSV (σ^2^ *_p_/*(σ^2^ *_p_*+ σ^2^ *_s_*) = 0.86, 95% CI = 0.53–1 and 0.91, 95% CI = 0.74–1, respectively), and most of the between-species variation for DObsSV (σ^2^ *_p_/*(σ^2^ *_p_*+ σ^2^ *_s_*)  = 0.72, 95% CI = 0.43–0.98). Therefore, most of the differences between species in viral titres can be explained either by the host phylogeny or the distance from the natural host.

Is it the distance from the natural host, or host phylogeny *per se*, that is most important in determining viral replication and persistence in a new host? To allow a direct comparison of these two effects, we calculated the expected amount of change in viral titre from the root to the tips of the tree that will result from the phylogenetic effect. This was done by taking the product of the standard deviation of the phylogenetic effect and 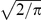, which is the mean of a folded zero-centred normal distribution, and is the predicted change under a Brownian model. This gave values of 2.15, 3.28 and 2.69 viral-titre-units for DAffSV, DMelSV and DObsSV respectively. These can be compared directly to the estimates described above of the amount of change in viral titre as the genetic distance from the natural host increases (−1.82, −0.47 and −3.70 viral-titre-units for DAffSV, DMelSV and DObsSV respectively). The time from the root to tip of the phylogeny has been estimated as ∼40 million years [60], so for every ∼40 million years travelled along the phylogeny, or from the natural host, we expect to see the above changes in viral titre. From these estimates it is clear that over this timescale the two processes are of similar importance for DAffSV and DObsSV, but that the host-phylogeny is more important than distance-from-the-original-host in determining the replication and persistence of DMelSV in a new host.

Differences between hosts in viral replication and persistence could either reflect differences in susceptibility to all three viruses (‘general susceptibility’), or the effects on the three viruses could be independent (‘specific susceptibility’). We found that most of the phylogenetic effect was caused by species differing in their level of general susceptibility, as there were strong phylogenetic correlations between viruses (Table 1). Furthermore, the correlation is not greater between the two viruses that naturally infect closely related hosts (DAffSV and DObsSV). Therefore, the phylogenetic effects mean that a given host species' susceptibility to one virus is strongly correlated to its susceptibility to another sigma virus, regardless of whether the virus originated from a closely or distantly related host.

**Table 1:** Phylogenetic correlations and 95% CI between each pair of viruses.

Viruses	Phylogenetic correlation *r*	95% CI
DAffSV-DObsSV	0.67	0.33–0.96
DAffSV-DMelSV	0.74	0.50–0.95
DObsSV-DMelSV	0.78	0.54–0.98

The analysis above assumes that we have the correct phylogeny, but some of the relationships are poorly resolved (Figure 1). To check whether this affected our results, we repeated the analysis integrating over the posterior sample of trees generated during the phylogenetic analysis [61]. This was achieved by fitting the phylogenetic mixed model to 2000 different trees from the posterior sample (from 100,000 trees we used a burn-in of 30,000 trees and then used every 35^th^ tree). This gave very similar results to our main analysis, suggesting that phylogenetic uncertainty does not affect our conclusions. We would note however, that σ^2^ *_p_* is biased downwards whenever the tree is incorrect, and this bias is not removed by this procedure.

## Discussion

We found that the ability of three sigma viruses to persist and replicate in 51 different species of *Drosophila* is largely explained by the host phylogeny. The effect of phylogeny can be broken down into two components; not only did viral titres tend to decline with increasing genetic distance from the natural host, but there is also a tendency for related hosts to have similar titres, independent of the distance effect.

The decline in viral titres with increasing distance from the natural host suggests that the greater the change in the cellular environment, the less well adapted the virus is. This might be caused by changes in the cellular machinery used by the virus in its replication cycle, or the virus being less adept at avoiding or suppressing the immune response. Regardless of the causes of this effect, it suggests that successful host shifts may be more likely between closely related hosts [6]. A host shift requires the new host to be exposed to the pathogen, the virus to replicate sufficiently for an individual to become infected, and finally for there to be sufficient onward transmission for the infection to become established in the population. Our data suggests that the second step is most likely to occur between closely related hosts. It is possible that higher titres may also lead to greater onward transmission, as the titre of DMelSV in *D. melanogaster* correlates with the rate at which the virus is transmitted [31], [62]. Furthermore, it has also been reported that although DMelSV will replicate in a range of *Drosophila*, but it was stably transmitted only in the closely related *Drosophila simulans* and not the more distantly related *Drosophila funebris* [63]. However, viral titres should only be used with caution as a proxy for transmission rates, as many other factors may affect transmission rates, including trade-offs between replication and virulence [64].

There is tentative evidence that host shifts of sigma viruses occur most often between closely related species in natural populations. Although comparisons of *Drosophila* and sigma virus phylogenies show evidence of past host shifts, the host and virus phylogenies are more similar than expected by chance [30]. This may be the result of more frequent host switches between closely related species, as would be predicted by our results (although cospeciation would produce the same pattern and more data is required to confirm these findings).

This result is interesting because it has previously been questioned whether the genetic distance between host species plays an important role in predicting the source of host shifts, especially for RNA viruses [6], [22]. Indeed, some plant viruses can replicate in an enormous range of species; Cucumber mosaic virus can infect 1,300 plant species in over 100 families and Tomato spotted wilt virus can infect 800 plant species in 80 families [65]. The use of conserved receptors to enter host cells may be key to large potential host ranges in animals [23], [24], [66]. However, although a virus may be able to enter the cells of many different species, it then relies on numerous different components of the cellular machinery to replicate effectively, and this may make shifts to hosts that are distant from the natural host unlikely.

A factor that could lead to changes in host suitability across the phylogeny is selection for resistance to viruses. One reason to suspect that this may be important is that genes involved in antiviral immunity often evolve exceptionally rapidly in *Drosophila* [67], [68], [69], [70], and this may translate into rapid phenotypic changes in host susceptibility. If this process is driving the patterns that we see, then the observation that natural host-parasite combinations tend to be more susceptible would suggest that the viruses have been able to overcome these host defences, resulting in viruses that are well adapted to their natural hosts, rather than *vice versa*.

After accounting for the effect of distance from the natural host, the host phylogeny still explains most of the remaining variation in viral titre between species. This ‘phylogenetic effect’ means that that closely related host species have similar levels of resistance due to their non-independence as a result of common ancestry. Indeed, the most distantly related clade to all of the natural hosts examined (the *Scaptodrosophila*) have one of the highest viral titres (Figure 1). For two of the viruses (DAffSV and DObsSV), we found that this phylogenetic effect was of comparable importance to the effect of genetic distance from the natural host, and for the third virus (DMelSV) it was more important.

The phylogenetic effect and distance effects are statistically (and biologically) distinct phenomena. If we imagine two sister species (A and B) and an out-group (C) are infected with a virus originally from species A, there are two ways in which the host phylogeny could affect the ability of the viruses to infect the three species. Under a Brownian motion model of evolution we expect viral titre in species A to be more different to that in C than B. Importantly, however, we do not expect this difference to have a consistent sign, as it is only the magnitude of the difference that should be larger for species C. A second process is that as we move away from species A we may expect a systematic change in viral titre – either that the viral titre increases as we move to species B and then to species C, or alternatively a systematic decrease. We call this first effect – where the change does not have a predictable sign – a phylogenetic effect, and the second effect - where change does have a predictable sign – a distance effect.

The phylogenetic and distance effects may also generate distinct patterns of host switching (see Introduction). For example, our data regarding the phylogenetic effect imply that sigma viruses may more easily switch between infecting flies in the subgenus *Sophophora* and the distantly–related, but highly susceptible, *Scaptodrosophila*. However, the two distinct patterns may emerge from the same underlying evolutionary process. If related hosts have similar levels of susceptibility (i.e. the phylogenetic effect), and pathogens can only become established in the most susceptible hosts, then we would expect to see a decline in viral titre in species distantly related to the natural hosts (i.e. the distance effect).

The phylogenetic effect is mostly caused by variation in susceptibility to all three viruses (there is a strong phylogenetic correlation in the titres of the three viruses). Such patterns may arise if the common ancestors of different host clades have acquired or lost immune or cellular components that affect susceptibility to all sigma viruses. The frequent gain and loss of immune components is well-established, for example, *Drosophila* species in the *obscura* group have lost a type of blood cell (lamellocytes) that are found in other *Drosophila*, which means they are particularly susceptible to parasitoid wasps [26]. Similarly a class of antifungal peptides (drosomycins) are found only in the *melanogaster* group of *Drosophila* [71], [72] and components of antiviral RNAi pathways have lineage-specific distributions [73], [74]. Part of the phylogenetic effect could be explained by the evolutionary history of the viruses, for example if they have recently switched between host species and are still well-adapted to a previous host. The strong phylogenetic correlation between the three viruses we studied might seem surprising as these viruses are very different to one another at the sequence level (amino-acid identities are ∼20%–40% [29], [30]). However, even viruses which show no similarities at the sequence level often share elements of protein structure [75], [76], [77], and different rhabdoviruses are known to have similar modes of action (for example, infecting nervous tissue [31], [78]).

The strong phylogenetic effect that we found also has practical implications for comparative studies of resistance in different species. It means that observations on related species will not be independent, so it is essential to account for these effects in the analysis of comparative data [79]. For example, the decline in the resistance of novel hosts with genetic distance from the natural hosts that has been observed in some previous studies may be attributable to a phylogenetic effect, rather than distance itself.

In conclusion, our results show that the host phylogeny is an important determinant of viral persistence and replication in novel hosts, and therefore may also be an important influence on the source of new emerging diseases. The effect is more subtle than simply leading to a decline in infection success with genetic distance from the original host, because the strong phylogenetic effect may sometimes result in susceptible hosts being grouped in phylogenetically distant clades, allowing parasites to jump great phylogenetic distances. The importance of these phylogenetic effects on replication and persistence relative to factors affecting exposure and onward transmission requires further study if we are to understand how they affect a parasites ability to host shift in nature.

## Supporting Information

Dataset S1 Alignment of *28S* sequences used in phylogenetic analysis in fasta formatted text file.
(TXT)

Dataset S2 Alignment of *Adh* sequences used in phylogenetic analysis in fasta formatted text file.
(TXT)

Dataset S3 Alignment of *Amyrel* sequences used in phylogenetic analysis in fasta formatted text file.
(TXT)

Dataset S4 Alignment of *COI* sequences used in phylogenetic analysis in fasta formatted text file.
(TXT)

Dataset S5 Alignment of *COII* sequences used in phylogenetic analysis in fasta formatted text file.
(TXT)

Dataset S6 Alignment of *RpL32/RP49* sequences used in phylogenetic analysis in fasta formatted text file.
(TXT)

Dataset S7 Alignment of *SOD* sequences used in phylogenetic analysis in fasta formatted text file.
(TXT)

Dataset S8 Accession numbers of sequences used in phylogenetic analysis in excel file.
(XLS)

Figure S1 A pilot study was used to measure the change in viral titre at fixed time points post-injection (0,1,3,5,10 days). Viral titre is measured relative to the amount injected (i.e. day 0). A large decrease in titre was found immediately after injection, with viral titre beginning to increasing again around 3–5 days post injection. The different coloured lines represent the different host species injected.
(TIFF)

Figure S2 Model estimates of distance effects for each virus (DAffSV is black, DMelSV is red, DObsSV is blue) with the different lines representing the posterior distribution estimated using the different priors (the solid line  =  prior 1 (inverse wishart), the dotted line  =  prior 2 (flat) and the dashed line  =  prior 3 (parameter expanded). Vertical lines are estimates of the distance effect from the ASREML analysis for each virus.
(TIFF)

Table S1 Full list of species used; whether they harboured Wolbachia (yes or no); their rearing temperature; whether they were composed of multiple lines (yes or no); food medium reared on (b = banana, l = lewis, lm = lewis with mushroom (peeled *Agaricus bisporus*), m = malt (recipe below), i =  4–24 instant Drosophila medium Carolina (Burlington, North Carolina, U.S.A.), im =  instant with mushroom), and mean wing length. All species are in the genus *Drosophila*, with the exceptions of; *Scaptomyza pallida, Hirtodrosophila duncani, Zaprionous badyi* and *Scaptodrosophila. lebanonensis* and *Scaptodrosophila. stonei*.
(DOC)

Table S2 *RpL32* primers for sequencing. Initially the *RpL32* seq F and R pair were used. However, if these failed, then combinations of the remaining primers were used. DNA was extracted using a Chelex-Proteinase K extraction and PCRs were carried out using a touchdown PCR cycle (95°C 30 sec, 62°C (−1°C per cycle) 30 sec, 72°C 1 min; for 10x cycles followed by; 95°C 30 sec, 52°C 30 sec, 72°C 1 min; for a further 25x cycles). In cases where the initial PCRs did not work, the PCR was repeated on cDNA. Following PCR, unincorporated primers and dNTPs were removed using exonuclease I and shrimp alkaline phosphatase, and the products were then sequenced in both directions using BigDye v3.1 (Applied Biosystems) and using a ABI capillary sequencer (Gene Pool facility, University of Edinburgh). The sequence chromatograms were inspected by eye to confirm the validity of all variants within and between species and assembled using Sequencher (v4.9).
(DOC)

Table S3 qRT-PCR primers. Drosophila *RpL32* primers were designed to match the homologous sequence in each species and crossed an intron-exon boundary so will only amplify mRNA. The intron location (located bases 457:518 in *D. melanogaster* accession: Y13939) was confirmed in a subset of 7 species (*D. melanogaster, D. obscura, D. affinis, D. paramelanica, D. ambigua, D. algonquin* and *Scaptomyza pallida*). Sigma virus primers crossed gene boundaries so as to only amplify genomes and not mRNA. We were unable to sequence *RpL32* for *D. busckii*. However, we found that the most closely related species in this study (*Z. badyi*) primers worked successfully in this species, with a suitable efficiency, and the PCR product was confirmed to be *RpL32* by sequencing.
(DOC)

Table S4 Drosophila gene sequencing primers for creating the phylogeny. PCRs were carried out using a touchdown PCR cycle (see Table S2) of 62–52°C for *COII* and *28s*, and 58–48°C for *COI, Adh* and *Amyrel*, then sequenced as described above (Table S2).
(DOC)

Text S1 Prior specification, fly food recipes and MCMCglmm syntax.doc.
(DOC)
