# Supplementary figures and images for: Host Phylogeny Determines Viral Persistence and Replication in Novel Hosts
Source: PLoS Pathog. 2011 Sep 22;7(9):e1002260. doi: 10.1371/journal.ppat.1002260 (PMC3178573; doi:10.1371/journal.ppat.1002260)

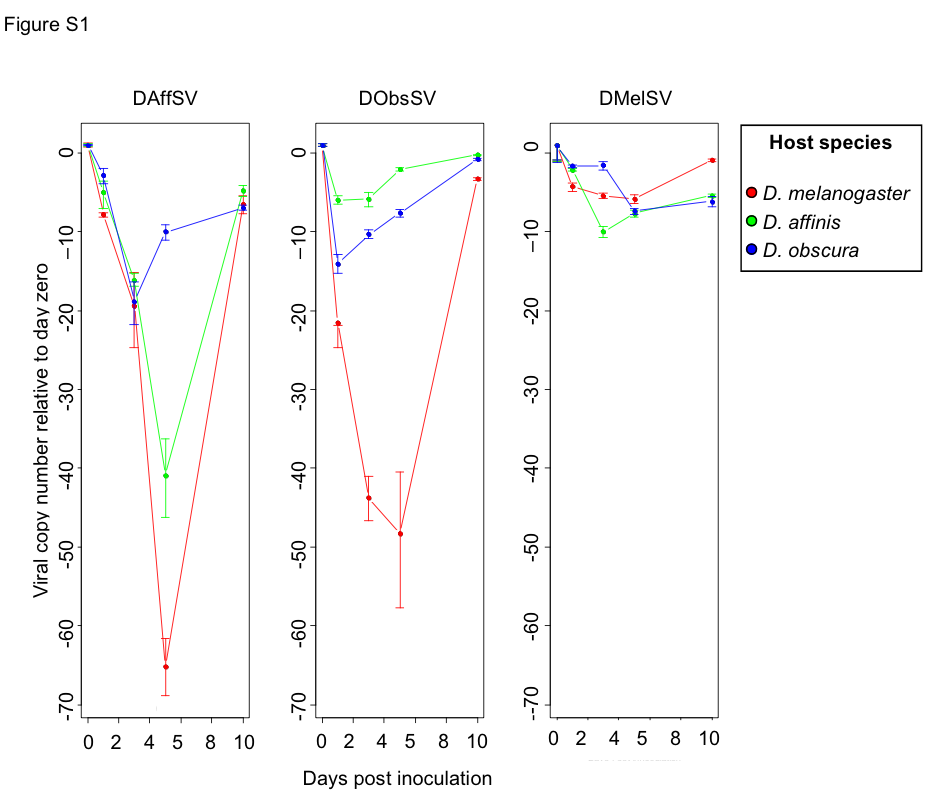

Supplement: Figure S1 — A pilot study was used to measure the change in viral titre at fixed time points post-injection (0,1,3,5,10 days). Viral titre is measured relative to the amount injected (i.e. day 0). A large decrease in titre was found immediately after injection, with viral titre beginning to increasing again around 3–5 days post injection. The different coloured lines represent the different host species injected. (TIFF) [file ppat.1002260.s009.tiff]

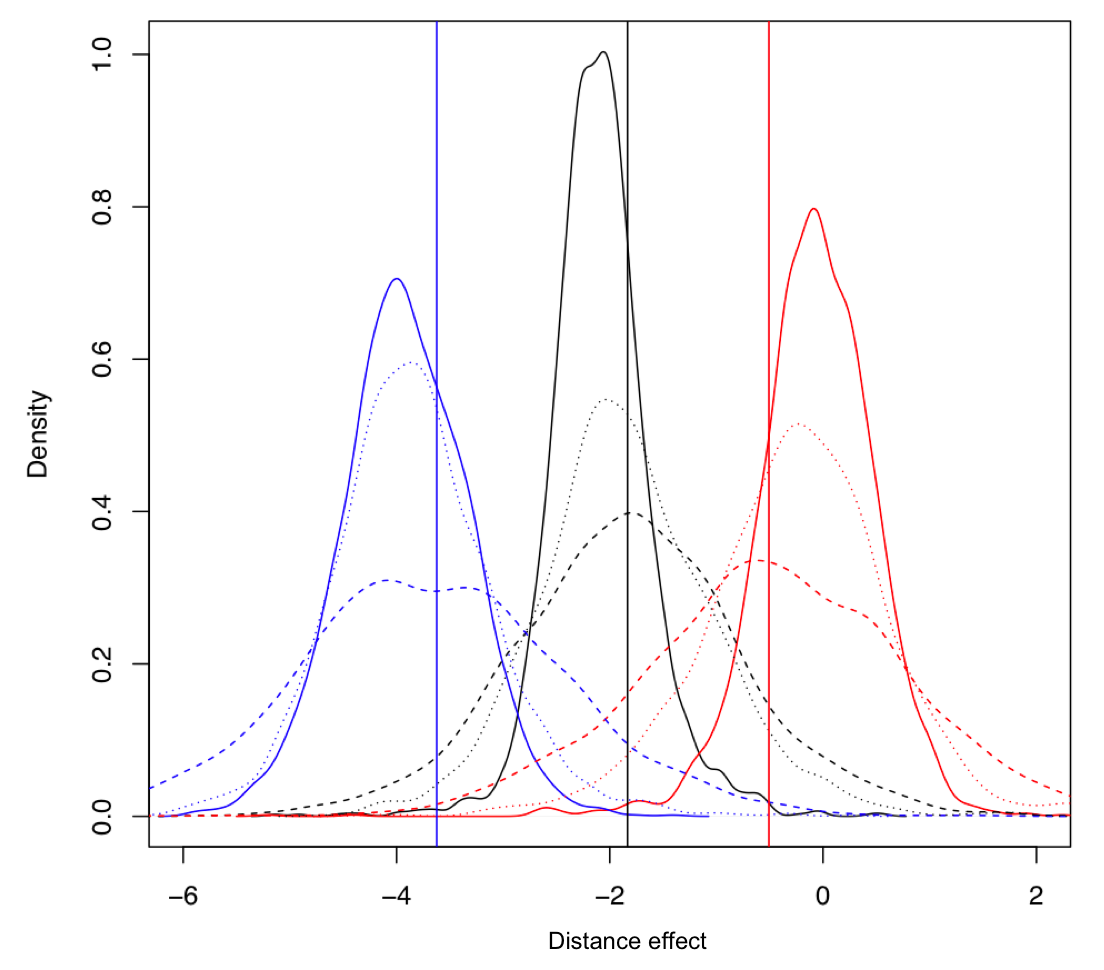

Supplement: Figure S2 — Model estimates of distance effects for each virus (DAffSV is black, DMelSV is red, DObsSV is blue) with the different lines representing the posterior distribution estimated using the different priors (the solid line = prior 1 (inverse wishart), the dotted line = prior 2 (flat) and the dashed line = prior 3 (parameter expanded). Vertical lines are estimates of the distance effect from the ASREML analysis for each virus. (TIFF) [file ppat.1002260.s010.tiff]
