# Supplementary material for: Host Phylogeny Determines Viral Persistence and Replication in Novel Hosts
Source: PLoS Pathog. 2011 Sep 22;7(9):e1002260. doi: 10.1371/journal.ppat.1002260 (PMC3178573; doi:10.1371/journal.ppat.1002260)
Supplement: Table S1 — Full list of species used; whether they harboured Wolbachia (yes or no); their rearing temperature; whether they were composed of multiple lines (yes or no); food medium reared on (b = banana, l = lewis, lm = lewis with mushroom (peeled Agaricus bisporus), m = malt (recipe below), i = 4–24 instant Drosophila medium Carolina (Burlington, North Carolina, U.S.A.), im = instant with mushroom), and mean wing length. All species are in the genus Drosophila, with the exceptions of; Scaptomyza pallida, Hirtodrosophila duncani, Zaprionous badyi and Scaptodrosophila. lebanonensis and Scaptodrosophila. stonei. (DOC) [file ppat.1002260.s011.doc]

**Table S1**

| Species name | Wolbachia | Rearing Temp (°C) | Multiple lines | Food | Mean wing length (mm) |
| --- | --- | --- | --- | --- | --- |
| *D.tristis* | y | 18 | n | lm | 2.53 |
| *D.busckii* | n | 18 | y | i | 1.71 |
| *D.melanogaster* | y | 18 | y | l | 2.12 |
| *D.subobscura* | n | 18 | y | lm | 2.32 |
| *D.simulans* | n | 18 | y | l | 1.84 |
| *D.ananassae* | y | 25 | n | m | 1.68 |
| *D.takahashii* | n | 18 | n | l | 1.90 |
| *D.pseudotakahashii* | y | 18 | n | l | 1.92 |
| *D.affinis* | n | 18 | y | b | 2.25 |
| *D.persimilis* | n | 18 | n | m | 2.35 |
| *D.pseudoobscura* | n | 18 | n | b | 2.25 |
| *D.miranda* | n | 18 | n | b | 2.61 |
| *D.buzzatii* | n | 18 | n | b | 1.97 |
| *D.nigromelanica* | n | 18 | y | b | 2.20 |
| *D.bifasciata* | n | 18 | n | m | 2.34 |
| *H.duncani* | y | 25 | n | m | 2.03 |
| *D.orena* | n | 18 | n | m | 1.81 |
| *D.borealis* | n | 18 | n | b | 2.37 |
| *D.paramelanica* | n | 18 | n | b | 2.52 |
| *D.guanche* | n | 18 | n | m | 2.17 |
| *D.immigrans* | n | 18 | y | m | 2.60 |
| *Z.badyi* | n | 18 | n | m | 2.18 |
| *S.lebanonensis* | n | 18 | n | m | 2.10 |
| *D.erecta* | n | 18 | n | m | 1.63 |
| *S.stonei* | y | 25 | n | m | 2.13 |
| *D.ambigua* | n | 18 | n | m | 2.36 |
| *D.algonquin* | n | 18 | n | m | 2.36 |
| *D.hydei* | n | 18 | n | m | 2.50 |
| *S.pallida* | n | 18 | n | i | 2.10 |
| *D.phalerata* | n | 18 | n | im | 2.57 |
| *D.tenebrosa* | n | 18 | n | im | 2.23 |
| *D.sechellia* | n | 18 | y | l | 1.66 |
| *D.nebulosa* | n | 18 | n | l | 1.93 |
| *D.santomea* | n | 18 | n | l | 1.75 |
| *D.lummei* | n | 18 | n | b | 2.81 |
| *D.lacicola* | n | 18 | n | m | 3.06 |
| *D.flavomontana* | n | 18 | n | b | 2.90 |
| *D.novamexicana* | n | 18 | y | b | 2.38 |
| *D.littoralis* | n | 18 | n | b | 2.60 |
| *D.americana* | n | 18 | n | m | 2.26 |
| *D.virilis* | n | 18 | y | b | 2.54 |
| *D.obscura* | n | 18 | y | lm | 2.42 |
| *D.mojavensis* | n | 18 | y | m | 1.90 |
| *D.lini* | n | 18 | n | m | 1.65 |
| *D.ohnishii* | n | 18 | n | m | 1.76 |
| *D.mauritiana* | n | 18 | n | m | 1.66 |
| *D.montana* | n | 18 | y | m | 2.71 |
| *D.teissieri* | y | 18 | n | m | 1.91 |
| *D.saltans* | n | 25 | n | m | 1.66 |
| *D.willistoni* | n | 25 | n | m | 1.69 |
| *D.yakuba* | n | 18 | y | m | 1.74 |

Table S1: Full list of species used; whether they harboured Wolbachia (yes or no); their rearing temperature; whether they were composed of multiple lines(yes or no); food medium reared on (b=banana [1], l=lewis [2], lm=lewis with mushroom (peeled *Agaricus bisporus*), m=malt (recipe below), i= 4-24 instant Drosophila medium Carolina (Burlington, North Carolina, U.S.A), im= instant with mushroom), and mean wing length. All species are in the genus *Drosophila*, with the exceptions of; *Scaptomyza pallida, Hirtodrosophila duncani, Zaprionous badyi* and *Scaptodrosophila. lebanonensis* and *Scaptodrosophila. stonei*. References:

1. Longdon B, Wilfert L, Obbard DJ, Jiggins FM (2011) Rhabdoviruses in two species of Drosophila: vertical transmission and a recent sweep. Genetics Advance online doi: 10.1534/genetics.111.127696.

2. Lewis E (1960) A new standard food medium. Drosophila information service 34: 117-118.
