# Supplementary material for: Host Phylogeny Determines Viral Persistence and Replication in Novel Hosts
Source: PLoS Pathog. 2011 Sep 22;7(9):e1002260. doi: 10.1371/journal.ppat.1002260 (PMC3178573; doi:10.1371/journal.ppat.1002260)
Supplement: Table S2 — RpL32 primers for sequencing. Initially the RpL32 seq F and R pair were used. However, if these failed, then combinations of the remaining primers were used. DNA was extracted using a Chelex-Proteinase K extraction and PCRs were carried out using a touchdown PCR cycle (95°C 30 sec, 62°C (−1°C per cycle) 30 sec, 72°C 1 min; for 10x cycles followed by; 95°C 30 sec, 52°C 30 sec, 72°C 1 min; for a further 25x cycles). In cases where the initial PCRs did not work, the PCR was repeated on cDNA. Following PCR, unincorporated primers and dNTPs were removed using exonuclease I and shrimp alkaline phosphatase, and the products were then sequenced in both directions using BigDye v3.1 (Applied Biosystems) and using a ABI capillary sequencer (Gene Pool facility, University of Edinburgh). The sequence chromatograms were inspected by eye to confirm the validity of all variants within and between species and assembled using Sequencher (v4.9). (DOC) [file ppat.1002260.s012.doc]

Table S2

| Primer name | Sequence 5’-3’ |
| --- | --- |
| *RpL32* seq F | ACAGGCCCAAGATCGTGAAGAAGC |
| *RpL32* seq R | CTCTTGAGAACGCAGGCGACC |
| *RpL32* seq F1 | AGACACTGGCGCWGTAAT |
| *RpL32* seq F2 | YAACTATMAAATTSCAGCTCC |
| *RpL32* seq F3 | AATGACSATTCGCCCAGCRTACMGG |
| *RpL32* seq R1 | TGCGCTKGTTGGADCCRTAACC |
| *RpL32* seq R2 | CGGTTCTGCATVARCARVACC |

Table S2: *RpL32* primers for sequencing. Initially the *RpL32* seq F and R pair were used. However, if these failed, then combinations of the remaining primers were used. DNA was extracted using a Chelex-Proteinase K extraction [1] and PCRs were carried out using a touchdown PCR cycle (95°C 30sec, 62°C (-1°C per cycle) 30sec, 72°C 1min; for 10x cycles followed by; 95°C 30sec, 52°C 30sec, 72°C 1min; for a further 25x cycles). In cases where the initial PCRs did not work, the PCR was repeated on cDNA. Following PCR, unincorporated primers and dNTPs were removed using exonuclease I and shrimp alkaline phosphatase, and the products were then sequenced in both directions using BigDye v3.1 (Applied Biosystems) and using a ABI capillary sequencer (Gene Pool facility, University of Edinburgh). The sequence chromatograms were inspected by eye to confirm the validity of all variants within and between species and assembled using Sequencher (v4.9).

Reference:

1. Longdon B, Wilfert L, Obbard DJ, Jiggins FM (2011) Rhabdoviruses in two species of Drosophila: vertical transmission and a recent sweep. Genetics Advance online doi: 10.1534/genetics.111.127696.
