# Supplementary material for: Host Phylogeny Determines Viral Persistence and Replication in Novel Hosts
Source: PLoS Pathog. 2011 Sep 22;7(9):e1002260. doi: 10.1371/journal.ppat.1002260 (PMC3178573; doi:10.1371/journal.ppat.1002260)
Supplement: Table S3 — qRT-PCR primers. Drosophila RpL32 primers were designed to match the homologous sequence in each species and crossed an intron-exon boundary so will only amplify mRNA. The intron location (located bases 457:518 in D. melanogaster accession: Y13939) was confirmed in a subset of 7 species (D. melanogaster, D. obscura, D. affinis, D. paramelanica, D. ambigua, D. algonquin and Scaptomyza pallida). Sigma virus primers crossed gene boundaries so as to only amplify genomes and not mRNA. We were unable to sequence RpL32 for D. busckii. However, we found that the most closely related species in this study (Z. badyi) primers worked successfully in this species, with a suitable efficiency, and the PCR product was confirmed to be RpL32 by sequencing. (DOC) [file ppat.1002260.s013.doc]

Table S3

| qRT-PCR primer name and location/species | Sequence 5’-3’ |
| --- | --- |
| DMelSV F (L gene-5’ trailer junction) | TTCAATTTTGTACGCGGAATC |
| DMelSV R (L gene-5’ trailer junction) | TGATCAAACCGCTAGCTTCA |
| DAffSV F (L gene-5’ trailer junction) | GCAGATGTATTAGTCTGTCCACG |
| DAffSV R (L gene-5’ trailer junction) | TGTGAGTCCAAACGAAAGGA |
| DObsSV F (N-P gene junction) | TGGTTTCGATGGGTTAGTGG |
| DObsSV R (N-P gene junction) | ATTGGACAATGGGTCAAAGC |
| *RpL32* qRT-PCR F (*D. melanogaster*) | TGCTAAGCTGTCGCACAAATGG |
| *RpL32* qRT-PCR R (*D. melanogaster*) | TGCGCTTGTTCGATCCGTAAC |

Supplementary table 3. qRT-PCR primers. Drosophila *RpL32* primers were designed to match the homologous sequence in each species and crossed an intron-exon boundary so will only amplify mRNA. The intron location (located bases 457:518 in *D. melanogaster* accession: Y13939) was confirmed in a subset of 7 species (*D. melanogaster, D. obscura, D. affinis, D. paramelanica, D. ambigua, D. algonquin* and *Scaptomyza pallida*). Sigma virus primers crossed gene boundaries so as to only amplify genomes and not mRNA. We were unable to sequence *RpL32* for *D. busckii*. However, we found that the most closely related species in this study (*Z. badyi*) primers worked successfully in this species, with a suitable efficiency, and the PCR product was confirmed to be *RpL32* by sequencing.
