# Supplementary material for: Host Phylogeny Determines Viral Persistence and Replication in Novel Hosts
Source: PLoS Pathog. 2011 Sep 22;7(9):e1002260. doi: 10.1371/journal.ppat.1002260 (PMC3178573; doi:10.1371/journal.ppat.1002260)
Supplement: Table S4 — Drosophila gene sequencing primers for creating the phylogeny. PCRs were carried out using a touchdown PCR cycle (see Table S2) of 62–52°C for COII and 28s, and 58–48°C for COI, Adh and Amyrel, then sequenced as described above (Table S2). (DOC) [file ppat.1002260.s014.doc]

**Table S4**

| Primer | Sequence 5’-3’ |
| --- | --- |
| *COI* seq F | ACAAATCAYAARGATATTGGAAC |
| *COI* seq R | TADCTRTGTTCAGCDGG |
| *COI* internal sequencing only F | TTTTGGNCAYCCWGAAGT |
| *Adh* seq F | GGYATTGGHYTSGACACCAG |
| *Adh* seq R | GARTCCCAGTGCTKGGTCCA |
| *COII* (provided by Greg Spicer) seq F | ATGGCAGATTAGTGCAATGG |
| *COII* (provided by Greg Spicer) seq R | GTTTAAGAGACCAGTACTTG |
| *28S* rDNA seq F | AGTTCAGCACTAAGTCAC |
| *28S* rDNA seq R | TTAGACTCCTTGGTCCGTG |
| *Amyrel seq* F | CAGCACAAYCCHCANTGGTG |
| *Amyrel seq* R | TGATGCCRTAKGGRWAGGCCA |

Supplementary table 4. Drosophila gene sequencing primers for creating the phylogeny. PCRs were carried out using a touchdown PCR cycle (see table S2) of 62-52°C for *COII* and *28s*, and 58-48°C for *COI, Adh* and *Amyrel*, then sequenced as described above (table S2).
