# Supplementary material for: Host Phylogeny Determines Viral Persistence and Replication in Novel Hosts
Source: PLoS Pathog. 2011 Sep 22;7(9):e1002260. doi: 10.1371/journal.ppat.1002260 (PMC3178573; doi:10.1371/journal.ppat.1002260)
Supplement: Text S1 — Prior specification, fly food recipes and MCMCglmm syntax.doc. (DOC) [file ppat.1002260.s015.doc]

**Text S1**

**Prior specification**

In a Bayesian analysis prior probability distributions have to be specified for the fixed effects and the covariance matrices **V**_p_, **V**_s_ and **V**_e_. Independent normal priors with zero mean and large variance (10^10^) were used for the fixed effects and are virtually non-informative in this context. An inverse-Wishart prior was placed on each diagonal element of **V**_e_ (i.e. the virus specific residual variances) with scale matrix and degree of belief parameter set to 0.002. This corresponds to an inverse-gamma distribution with shape and scale parameters set to 0.001 and is weakly informative when the posterior distribution does not have substantial density at values close to zero, which was the case.

Diffuse priors for fully parameterised covariance matrices such as **V**_p_ and **V**_s_ are harder to define so we used several types of prior with differing properties, in order to evaluate the sensitivity of our conclusions to alternative prior specifications. Historically inverse-Wishart priors with low degree of belief had been used as purported weakly informative prior distributions because they are conjugate to the likelihood for covariance matrices. Conceptually, having a degree of belief of nu is like observing nu effects (a priori) with known variance which is specified (V). More recently, it has been suggested that inverse-Wishart priors (which are equivalent to inverse-gamma priors when the covariance matrix is 1x1 and simply a variance) can be highly informative when the posterior distribution has some support at small values, and parameter expanded priors have been suggested as a good alternative [1]. Parameter expansion results in F priors (or t priors for the standard deviation), and also improves mixing of the chain. We evaluated our posterior distribution under several commonly used priors:

prior 1: inverse-Wishart with scale = **I***0.002 and degree of belief = 2.002 (i.e V=diag(3)*(0.002/2.002), nu=2.002) which induces a marginal prior distribution on the variances that is equivalent to an inverse-gamma distribution with shape and scale equal to 0.001.

prior 2: a flat prior (i.e V=0, nu=0) which for 3x3 covariance matrices results in posterior modes that should coincide with the REML estimates [2].

prior 3: a parameter expanded prior was used with scale = **I**, degree of belief = 3 and a multivariate normal prior specification for the three redundant working parameters with null mean vector and covariance **I***10^3^ (i.e V=diag(3)/3, nu=3, alpha.mu=rep(0,3), alpha.V=diag(3)* 10^3^). This induces a scaled F-distributed marginal prior on the variances with numerator and denominator degrees of freedom equal to 1 and scale set to 10^1.5^ (see [1] for details).

Posterior modes for the species-level variances were close to zero for viruses A and M and these were omitted from the model and a species-level variance estimated for virus O only. In this case the prior for the single variance was:

prior 1: scale=0.002 and degree of belief =0.002 (i.e. V=1, nu=0.002)

prior2: V=0, nu=-2

prior3: scale=1, degree of belief =1, and null mean and variance 10^3^ for the working parameters (i.e. V=1, nu=1, alpha.mu=0, alpha.V=1000)

Parameter expanded models were run for 500,000 iterations with a thinning interval of 200 and a burn-in of 50,000, whereas all non parameter expanded models were run for 10 times as long due to their poorer mixing properties. This resulted in 2250 samples from the posterior for which autocorrelation between successive samples for all parameters was less than 0.01. It has been suggested that parameter expanded priors are preferable when weakly informative priors are sought [1] and indeed they gave point estimates consistent with the REML analysis (Figure S2) and so are those presented (they also have the lowest Deviance Information Criterion (DIC)). All analysis gave very similar results.

**Fly food recipes**

Malt food recipe: 1000ml distilled water, 10g agar, 60g semolina, 20g yeast, 80g powdered malt extract, brought to the boil and simmered for 15 mins, cooled for 20mins and then 14ml nipagin (10%) and 5ml proprionic acid added.

Agar food for ageing post injection: 1000ml water, 20g agar, 84g sugar-boiled, cooled then add 7mls tergosept.

**MCMCglmm syntax (R script) for priors and main model**

prior1<-list(G=list(G1=list(V=diag(3)*(0.002/2.002),n=2.002), G2=list(V=diag(3)*(0.002/2.002),n=2.002)), R=list(V=diag(3),nu=0.002))

prior2<-list(G=list(G1=list(V=diag(3)*1e-2,nu=1e-2), G2=list(V=diag(3)*1e-2,nu=1e-2)), R=list(V=diag(3)*1e-2,n=1e-2))

prior3<-list(G=list(G1=list(V=diag(3),n=3, alpha.mu=rep(0,3), alpha.V=diag(3)*1000), G2=list(V=diag(3),n=3, alpha.mu=rep(0,3), alpha.V=diag(3)*1000)), R=list(V=diag(3),n=0.002))

#Alterations to priors so only species level variance for DObsSV (coded as “O”)

prior1$G$G2<-list(V=1, nu=0.002)

prior2$G$G2<-list(V=1e-8, nu=-2)

prior3$G$G2<-list(V=1, nu=1, alpha.mu=0, alpha.V=1000)

model <-MCMCglmm(change_in_viral_titre~distance*virus, random=~us(virus):animal+us(at.level(virus, "O")):fly_species, rcov=~idh(virus):units, data=dataset, prior=prior3, pedigree=tree, nitt=500000, thin=200, burnin=5000)

**Supporting References**

1. Gelman A (2006) Prior distributions for variance parameters in hierarchical models. Bayesian Analysis 1: 515-533.

2. Sorensen D, Gianola D (2002) Likelihood, Bayesian and MCMC Methods in Quantitative Genetics. New York: Springer-Verlag.
